# Pharmacist interventions in Asian healthcare environments for older people: a systematic review and meta-analysis on hospitalization, mortality, and quality of life

**DOI:** 10.1186/s12877-024-05089-9

**Published:** 2024-06-12

**Authors:** In-Ja Kim, Gina Ryu, Sandy Jeong Rhie, Hwa-Jung Kim

**Affiliations:** College of Pharmacy and Graduate School of Pharmaceutical Sciences, Research Institute of Pharmaceutical Science, Ewha Womans University, 52 Ewhayeodae-Gil, Seodaemun-Gu, Seoul, 03760 Republic of Korea

**Keywords:** Asia, Hospitalization, Mortality, Quality of life, Older people, Pharmacist intervention

## Abstract

**Background:**

Pharmaceutical interventions play a key role in the care of older people experiencing polypharmacy. Despite the rapid increase in the aging population in Asia, there is a lack of evidence regarding the effectiveness of pharmacist interventions on older adult’s healthcare. This systematic review and meta-analysis assessed the effects of pharmacist interventions in Asian health care environments on hospitalization, mortality, and quality of life (QoL) among older people in Asia.

**Methods:**

A comprehensive search was conducted across 5 databases, encompassing studies published from inception through June 2023. Only studies involving pharmacist interventions for people aged 65 years or older, residing in Asian countries, were considered. Studies without evidence of pharmacist involvement or conducted outside of Asia were excluded. Data extraction was performed by two reviewers, one reviewer (I.K.) performed the initial extraction, and another reviewer (G.R.) verified the extracted data. Forest plots were generated using a random effects model to obtain risk ratios or pooled standardized mean differences (SMDs).

**Results:**

A total of 170 articles underwent thorough review, and ultimately, ten studies meeting the inclusion criteria were included in the meta-analyses. These studies encompassed diverse healthcare settings such as outpatient, inpatient, and nursing homes, with sample sizes ranging from 32 to 306 older people. Pharmacist interventions were found to significantly reduce hospitalization rates (*n* = 5, risk ratio = 0.57, 95% CI = 0.41–0.81) and mortality rates (*n* = 4, risk ratio = 0.57, 95% CI = 0.37–0.88) among older people. The analysis revealed less significant improvement in QoL in these patients than in those receiving usual care (*n* = 6, SMD = 0.36, *P* = 0.057).

**Conclusions:**

These findings highlight the crucial role of pharmacists within healthcare teams in Asian countries. Pharmacist interventions have an impact on reducing hospitalization and mortality rates among the elderly people, underscoring the importance of optimizing patient outcomes in Asia.

**Supplementary Information:**

The online version contains supplementary material available at 10.1186/s12877-024-05089-9.

## Background

The global population is aging, necessitating the development of appropriate healthcare systems to address the needs of rapidly aging populations [1]. One common issue among older people is polypharmacy, which refers to the intake of five or more medications due to the presence of multiple chronic diseases. Recent reports suggest polypharmacy reduces health-related quality of life (HRQOL) and raises risks of potentially inappropriate medications (PIMs) linked to severe side effects, including hospitalization or death [2–4].

Pharmacists are becoming increasingly important in managing the challenges of polypharmacy [5]. Recently, clinical medication review (CMR) and deprescribing have been widely used as interventions to solve polypharmacy problems [6]. CMR was further divided into three types according to their purpose: prescription-only review (CMR1), adherence review (CMR2), comprehensive medication review considering patient’s condition (CMR3) as described in previous reports [7, 8]. Deprescribing, defined as “the process of withdrawal of an inappropriate medication, supervised by a health care professional with the goal of managing polypharmacy and improving outcomes” [9] is a strategy that reduces unnecessary medications to patients with four or five steps, and is being tried in various setting environments in solving polypharmacy [10–12]. Medication reconciliation (MR) is a type of medication review that is performed in an inpatient setting [13]. Pharmacists compare patient's usual medications prior to admission to the prescribed medications they received upon admission to identify differences [13]. Multidisciplinary team (MDT) care is also emphasized because older people often have multiple chronic conditions, requiring a comprehensive care approach [14]. The types and characteristics of pharmacist interventions are summarized in Table 1.

**Table 1:** Type of pharmacist interventions

Type of pharmacist intervention		Pharmacist activity
CMR	CMR1	prescription-only review
	CMR2	adherence review
	CMR3	comprehensive medication review considering patient’s condition
Medication reconciliation (only inpatients)		compare patient's usual medications prior to admission to the prescribed medications they received upon admission to identify differences
MDT care		collaborate with specialists in your field to assess the patient's condition and find the optimal medication regimen
Deprescribing		the process of withdrawal of an inappropriate medication, supervised by a health care professional with the goal of managing polypharmacy and improving outcomes
Patient educations		Explain to patients how to use the medication and the dose to improve adherence

*Abbreviations*: *CMR* Clinical medication review, *MDT* Multidisciplinary team

The aging population in Asia is growing even more dramatically, with the proportion of people aged > 65 years of age expected to nearly double from 10% in 2022 to 19% in 2050 [1]. In Asia, pharmaceutical care varies depending on location, legal framework, political context, and healthcare system [15]. In middle- and lower-income countries (LMICs), where the number of physicians is known to be absolutely insufficient, task-sharing intervention, which complements the role of physicians by working as a team of health professionals, is gaining prominence [16]. Pharmacists' involvement in task-sharing has demonstrated improved clinical outcomes for patients with conditions like diabetes, hypertension, asthma, and even ovarian cancer, thanks to early detection of medication-related issues [16–19].

Several systematic review studies have examined the effects of pharmacist interventions on hospitalization, mortality, and quality of life (QoL) for older people, yielding inconsistent results. A recent review reviewed systematic reviews and meta-analyses of medication interventions for polypharmacy in older people [20]. Of the four studies that reported on hospitalization rates, two reported a reduction in hospitalization, while two reported no significant difference [8, 21–23]. Similar observations were made in studies conducted in the Organization for Economic Cooperation and Development (OECD) countries, where pharmacist interventions did not lead to significant changes in the unplanned admissions of older people [24]. This trend was also observed in an earlier study, which focused primarily on the United Kingdom and the United States [25]. However, a systematic review and meta-analysis conducted in the United States revealed overall positive effects of pharmacist interventions for geriatric patients, not only in terms of hospitalization but also regarding therapeutics, safety, and adherence outcomes [26]. Additionally, comprehensive medication reviews performed by pharmacists were found to reduce the risk of unplanned hospitalizations [8] and emergency department visits [23] among older people with polypharmacy. Regarding mortality outcomes, previous systematic reviews have shown either no significant effect [21, 25] or a considerable reduction as a result of pharmacist interventions [27, 28]. Positive effects on QoL outcomes were reported in one meta-analysis study but not in another [28, 29].

Although reports of advanced clinical pharmacy services in Asia are increasing, there is a shortage of research on the impact of pharmacist interventions on clinical outcomes in older people and review articles comparing pharmacist interventions in Asia to those in Europe or the West. Therefore, in this study, we aimed to evaluate the impact of pharmacist interventions on older people residing in Asia, particularly concerning hospitalization, mortality, and QoL through a meta-analysis.

## Methods

This systematic review was reported in compliance with the Preferred Reporting Items for Systematic Reviews and Meta-Analyses (PRISMA) guidelines [30]. A PRISMA checklist can be found in Additional file 1 & 2. The protocol was registered with PROSPERO (CRD42023388627).

### Eligibility criteria

A comprehensive literature search was conducted using five databases-PubMed, Embase, the Cochrane Central Register of Controlled Trials (CENTRAL), the Korean Medical Database (KMBASE), and the Research Information Sharing Service (RISS)-from their inception up to June 30, 2023. The search strategy utilized various combinations of the keywords 'pharmacist' and 'older people'. In PubMed, additional search terms included older adults, old people, old*, older people, older persons, elderly, senior, frail, aged, geriatric, geriatric patient, elderly patient, and pharmacist. The searches were limited to titles and abstracts. Additionally, the reference lists of relevant review articles and meta-analyses were manually searched to identify any potentially overlooked studies.

For inclusion in this study, the selected studies were required to evaluate the effectiveness of pharmacist interventions on all-cause hospitalization, readmission, unplanned hospital admission, mortality, and QoL in people aged 65 years or older. The specific outcome measures varied depending on the type of outcome being assessed. Studies that provided sufficient data to calculate risk ratios or standardized mean differences (SMDs) along with their corresponding 95% confidence intervals (CIs) were included. The study design was not limited to randomized controlled trials (RCTs) and encompassed studies that demonstrated evidence of pharmacist intervention, with a comparator group [31]. Only studies conducted in health care settings in Asia and reported in English were considered. The determination of whether a study was conducted in Asia was based on the United Nations's global regional grouping data [32]. Asia included countries corresponding to the regions of Western Asia, Central and Southern Asia, and Eastern and South-Eastern Asia [32].

### Study selection and data extraction

To ensure that only studies conducted in Asian healthcare settings (hospitals and nursing homes) were included, one reviewer (G.R.) screened the country of the healthcare settings where pharmaceutical care services were provided. Studies conducted outside of Asia were eliminated at this stage. Subsequently, two reviewers (G.R. and I.K.) independently screened the titles and abstracts of the remaining studies to identify those that required full-text reading. The final selection of papers was determined through discussions between the reviewers, and in cases where disagreements arose, a third reviewer (H.-J.K.) served as an arbitrator.

For the data extraction, one reviewer (I.K.) performed the initial extraction, and another reviewer (G.R.) verified the extracted data. In cases where the extracted data were incomplete or unavailable in a usable form, attempts were made to contact the authors of the selected papers to obtain the necessary information.

### Risk of bias assessments

The risk of bias in the included studies was evaluated using ROB2 tool for RCTs and ROBINS-I tool for non-RCTs [33, 34]. The judgement consisted of ‘low risk of bias,’ ‘some concerns,’ and ‘high risk of bias’. Two independent reviewers (I.K and G.R) conducted the risk of bias assessments for each study. In cases where inconsistencies arose, a third reviewer (H.-J.K.) served as an arbitrator to resolve any discrepancies.

### Data synthesis

To synthesize the results, we calculated the estimated effect of pharmacist interventions using the risk ratio for dichotomous outcomes such as hospitalization and mortality, and the SMDs for continuous outcomes such as QoL. The magnitude of an SMD was categorized as small (< 0.20), moderate (0.20–0.80), or large (> 0.80) based on evaluation guidelines [35]. Considering the diversity of interventions across studies, we constructed a forest plot using a random-effects model for data synthesis. In addition, subgroup analysis was conducted according to the healthcare setting of elderly patients to account for clinical diversity. The study design, setting, participant information (sample size, mean age, percentage of females, and inclusion criteria), intervention type, and outcome measures used in the meta-analysis were tabulated.

To assess study heterogeneity, we utilized the I^2^ statistic. In cases where high heterogeneity was observed, a sensitivity analysis was conducted by excluding studies that were deemed to be potential sources of heterogeneity like as studies with high risk of bias. Given that the number of included papers in this study was ≤ 10, publication bias was not assessed [36]. The data analysis was performed using the Comprehensive Meta-analysis Program software (Biostat, USA). A *p*-value of < 0.05 indicated statistical significance, and the results are reported with 95% confidence intervals (CIs).

The certainty of evidence was evaluated using Grading of Recommendations, Assessment, Development and Evaluation (GRADE) approach [37]. Each GRADE domain (imprecision, inconsistency, risk of bias, indirectness, and publication bias) was assessed and overall GRADE quality was rated as ‘very low’, ‘low’, ‘moderate’, and ‘high’.

## Results

### Search results

**Fig. 1:**
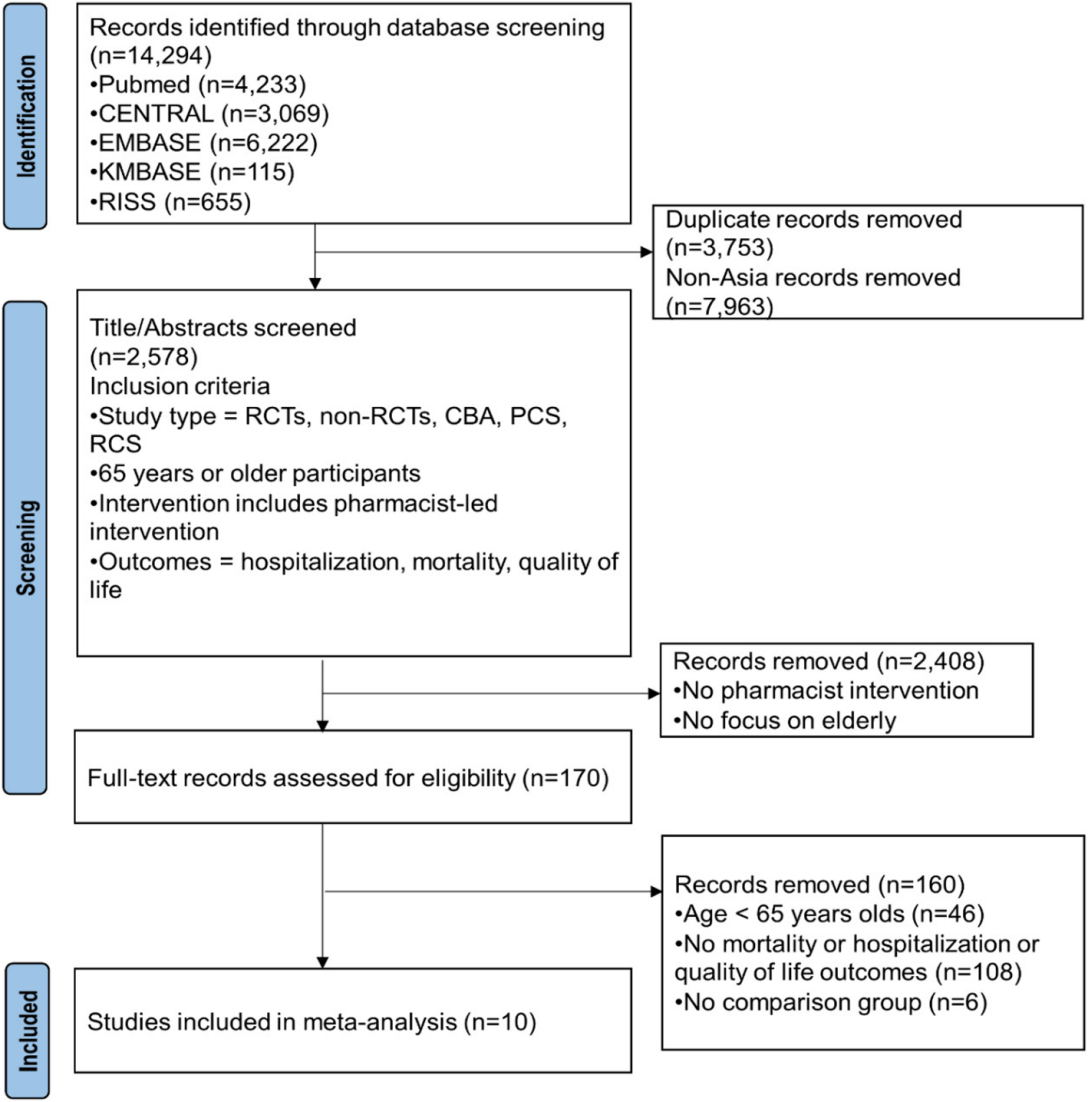
PRISMA diagram for the meta-analysis

After removing non-Asian studies and duplicates, a total of 2,578 studies were identified. The titles and abstracts of these studies were reviewed to determine their eligibility, resulting in 170 full-text studies being assessed for inclusion in this systematic review. Following the full-text review, a total of ten studies that met the inclusion criteria were selected and included in this review. The study selection process is illustrated in Fig. 1.

### Characteristics of the included studies

Table 2 provides an overview of the ten studies included in the present study. These studies were conducted in different regions, including China (*n* = 2) [38, 39], Hong Kong SAR (*n* = 1) [40], Israel (*n* = 1) [41], Japan (*n* = 2) [42, 43], Singapore (*n* = 1) [44], and Taiwan (*n* = 3) [45–47]. Among them, six studies utilized an RCT design, while four studies employed a non-RCT design. The study populations consisted of outpatients receiving treatment for chronic diseases or taking multiple medications (*n* = 4), older people residing in nursing homes (*n* = 4), and inpatients (*n* = 2). The outcome data from the eight included studies, which were used in the meta-analysis, are summarized in Table 2

**Table 2:** Characteristics of the included studies

Country	Author(year) Study design	Setting	Inclusion criteria	No. of patients (% Female)	Age in years mean (SD)	Outcome data used in meta-analysis
**China**	Zhang 2022 Non-RCT	Outpatient	Taking ≥ 5 drugs, with ≥ 1 chronic disease	I: 412 (53.2) C: 412 (53.2)	I: 73.43 (7.8) C: 73.43 (7.8)	EQ-5D index, mean change (sd): I = 0.78 (0.08), C = 0.75 (0.10)
	Zheng 2022 RCT	Inpatient	Patients who underwent elective orthopedic joint surgery, taking ≥ 1 drugs	I:33 (20) C:32 (16)	I: 67.4 (4.5) C: 68.2 (5.8)	Readmission within 30 Days (%): I = 0.0, C = 6.3
**Hong Kong** **SAR**	Chiu 2018 Non-RCT	Inpatient	Patient ≥ 65 years old	I: 107 (50) C: 101 (53.8)	I: 83.3 (5.7) C: 83.3 (5.6)	Unplanned hospital readmission at 1 months (%): I = 13.2, C = 29.1; Death at 3 months (%): I = 2.8, C = 4.9
**Israel**	Frankenthal 2014 RCT	Nursing home	Taking ≥ 1 drugs	I: 160 (70.5) C: 146 (62.5)	Total: 82.7 (8.7)	Hospital admission (%): I = 50, C = 50; Death at 1 years (%): I = 8.2, C = 9.7; SF-12 PCS, mean (sd): I = 33.1 (8.1), C = 33 (8.3)
**Japan**	Hashimoto 2020 Non-RCT	Nursing home	Taking ≥ 5 drugs	I: 28 (78.6) C: 27 (77.8)	I: 86.8 (7.1) C: 84.9 (7.4)	SF-12 PCS, mean (sd): I = 33.1 (8.1), C = 33 (8.3)
	Sakakibara 2015 Non-RCT	Outpatient	Dementia	I: 19 (21.1) C: 13 (23.1)	I: 88.3 (8.4) C: 83.7 (8.0)	EQ-5D index, mean change (sd): I = -0.03 (0.3), C = -0.13 (0.3)
**Singapore**	Kua 2021 RCT	Nursing home	Taking ≥ 5 drugs	I: 153 (89) C: 142 (75)	I: 80.57 (9.42) C: 80.02 (9.58)	Death at 1 years (%): I = 2.4, C = 5.3
**Taiwan**	Chen 2016 RCT	Outpatient	DM and HbA1c ≥ 9.0%	I: 50 (50) C: 50 (50)	I: 72.16 (6.6) C: 72.76 (5.9)	Hospital admission (%): I = 14, C = 32
	Lin 2018 RCT	Outpatient	Taking ≥ 6 drugs, with ≥ 3 chronic diseases, ≥ 4 visits for clinic	I: 87 (41.4) C: 91 (35.2)	I: 77.9 (6.1) C: 78.4 (6.0)	Death at 1 years (%): I = 2.3, C = 8.8; EQ-5D index, mean change (sd): I = 0.216 (0.2), C = -0.01 (0.2)
	Liou 2021 RCT	Nursing home	Taking ≥ 5 drugs, with ≥ 2 chronic diseases	I: 50 (50) C: 50 (50)	I: 86.7 (5.6) C: 85.7 (3.6)	EQ-VAS, mean change (sd): I = -7.5 (24.6), C = 1.2 (24.4)

*Abbreviations*: *C* Control group *DM* diabetes mellitus, *HbA1c* Hemoglobin A1c, *I* intervention group, *RCT* Randomized controlled trials, *SD* Standard deviation, *EQ-5D* European Quality of Life-5 Dimensions, *EQ-VAS* European quality of life-visual analog scale, *QoL* quality of life, *SF-12 PCS* 12-item Short-Form Health Survey Physical Component Summary

The selected papers described various types of pharmacist interventions, which were categorized into five types: clinical medication review (CMR), deprescribing, multidisciplinary team (MDT) care, medication reconciliation (MR), and patient education. Detailed information on the types of pharmacist interventions and pharmacist activity are presented in Table 3.

**Table 3:** Pharmacist intervention type and activity for each outcome

Outcome	Author (year)			Intervention type	Pharmacist activity						
					**How to**			**Information sources**			**Interventions** **for inappropriate medications**
**Hospitalization**	Chiu (2018)			CMR3, MR, patient education	MR-compare a patient's usual medications prior to admission to the prescribed medications they received upon admission to identify differences; CMR3- check for medication appropriateness using medication appropriateness index (MAI) on admission and at discharge; patient education- educate patients about the medication's indications, how to use it, side effects to watch out for, and how to store it. Provide a patient information leaflets			Electronic patient record; Patient's ward case notes; Interview with patient and/or patient carer			Primary electronic notification to patient's physician and verbal notification if strong intervention is warranted
	Chen (2016)			CMR3, MDT care, patient education	CMR3- medication adherence, dementia screening, and depression screening were conducted to review medication-related issues considering the patient's condition MDT- the patient was referred to a diabetes nurse or dietitian as needed to ensure proper diabetes care Patient education- patients were counseled on medication via outpatient visits or telephone and monitored monthly			Laboratory data were collected from the computerized physician order entry system; Interview with patient and/or patient carer			If there is a change in medication regimen after patient counseling, the prescription is changed with the doctor's confirmation
**Hospitalization**	Frankenthal (2014)			CMR1		CMR1-medication review by pharmacist for all residents at study beginning and 6 and 12 months later. STOPP criteria were applied to identify PIPs and PPOs	NR			Discussed with the chief physician at study beginning and after 6 months	
	Kua (2021)			CMR3, MDT care, deprescribing		MDT care- pharmacists, physicians, and nurses CMR3, deprescribing- 1) reviewing the necessity of medication using Beers and STOPP criteria; 2) checking for drug interaction to reduce risks of adverse drug events; 3) discussion with nurses on the feasibility of deprescribing; 4) communication through nurse to physician for reviewing and deprescribing decisions; 5) documentation	Medical record,			Communication through nurse to physician for reviewing and deprescribing decisions	
	Zheng (2022)			MDT care, MR, patient education		MDT care- physicians, nurses, pharmacist MR- medication review by pharmacist within 24 h of admission; collecting the medication history and laboratory data, and interview patients to obtain their medication history; compared with admitting medication orders; check discrepancies	Laboratory data; Interview with patient and/or patient carer			For unintended discrepancies, pharmacist conduct discontinue medication, add medication, continue at different doses/frequencies/routes/manufactures of medication, or substitute with a different medication. A new reconciliated medication list was formed and shared it with physician	
**Mortality**		Chiu (2018)	CMR3, MR, patient education			as above	as above			as above	
		Frankenthal (2014)	CMR1			as above	as above			as above	
		Kua (2021)	CMR3, MDT care, deprescribing			as above	as above			as above	
		Lin (2018)	CMR3, MDT care, patient education			MDT care- included 2 geriatricians, 1 cardiologist, 1 nephrologist, and 1 clinical pharmacist supervisor, 1 the study’s responsible clinical pharmacist CMR3- identifying MRPs such as medication duplications, drug interactions, dosing for renal and liver impairment, suspected ADEs; Patient education- face to face and telephone counseling				Bi-weekly team meetings, patient-centered discussions between the clinical pharmacist and physician	
**Quality of life**		Frankenthal (2014)	CMR1			as above	as above			as above	
		Hashimoto (2020)	CMR3, MDT care			MDT care- physician, nurse, and care worker CMR3: visiting nursing home once a week; check for residents with changes in medication, physical conditions, ADEs; check for resident eating, excretory, sleep and locomotion status, and treatment compliance, look for problems; share information with physician, briefing until next visit	Flow chart, nurse records, worker record			Discussion with teams and share with physicians	
**Quality of life**		Lin (2018)	CMR3, MDT care, patient education			as above	as above		as above		
		Liou (2021)	CMR1, patient education			CMR1- identifying DRPs Patient education- to provide accurate knowledge related to administration of medication	Medical chart notes		Medication recommendations for DRPs were conveyed to physicians by medical chart notes		
		Sakakibara (2015)	Deprescribing			Deprescribing- including the list of drugs requiring particular care in administration for elderly patients, utilization of combination drugs, simplification of administration, and utilization of orally disintegrating tablets			Intervention groups were reduced prescription drugs by the proposal made by pharmacist		
		Zhang (2022)	CMR3, patient education			CMR3- collect medication history and clinical information; identify DRPs based on the PCNE DRP classification; propose interventions to physicians or patients; assess medication adherence and HRQoL and discuss strategies with the patient Patient education—evaluate the patient’s understanding of medications and diseases and provide appropriate targeted education; schedule necessary follow-up appointments	Patients’ demographic and clinical information were obtained through interviews and their medical records				

*Abbreviations*: *ADE* Adverse drug event, *CMR 1,2,3* clinical medication review type1,2,3, *DRP* Drug-related problem, *HRQoL* Health-related quality of life, *MDT* Multidisciplinary team, *MR* Medication reconciliation, *NR* Not reported, *STOPP* Screening tool of older person’s prescriptions

### Risk of bias

**Fig. 2:**
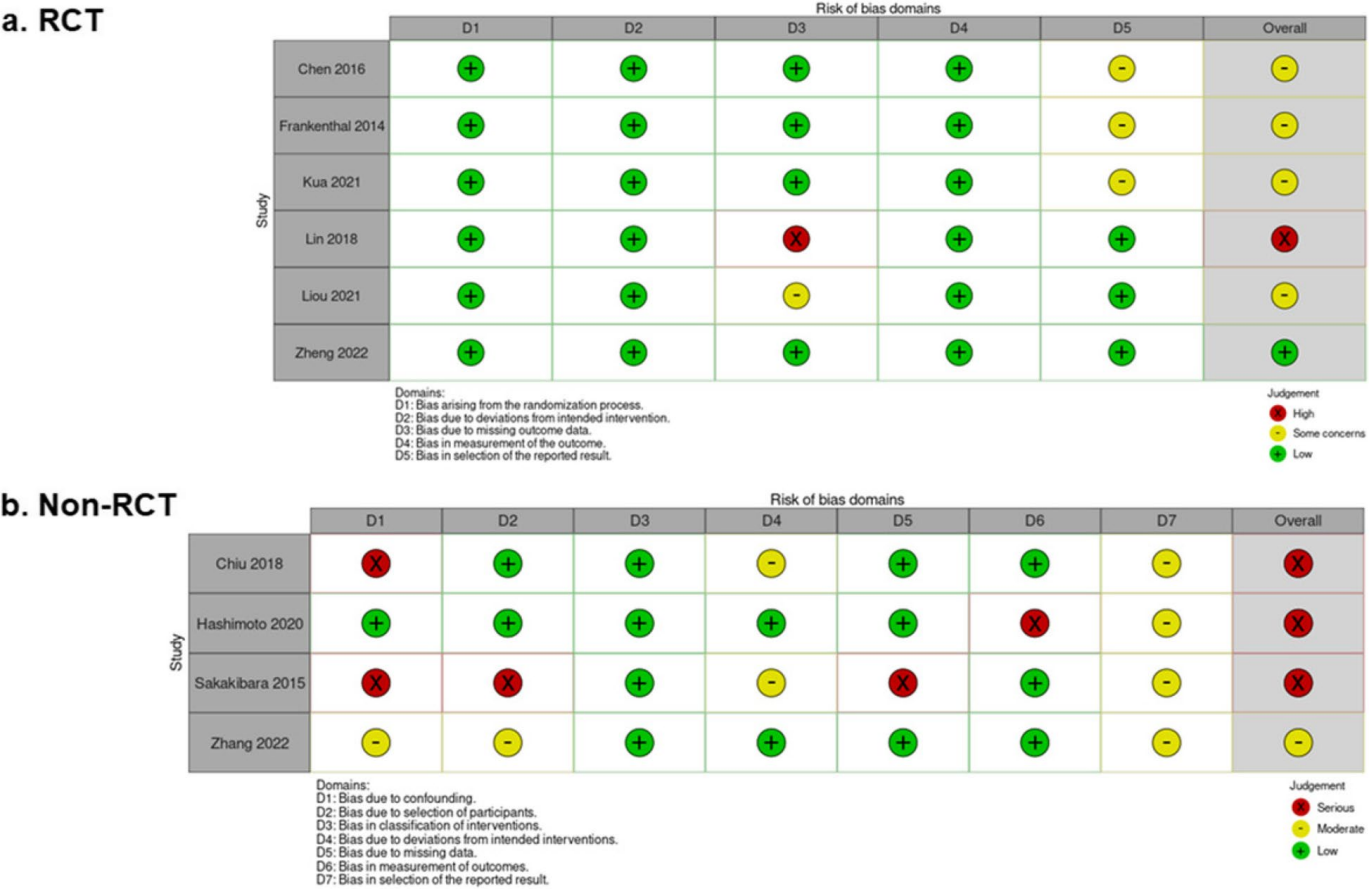
Risk of bias in the included study

Assessment of the risk of bias is summarized in Fig. 2. In six RCTs, one was rated as high risk [46], one as low risk [39], and the remaining four as uncertain [41, 44, 45, 47]. In four non-RCTs, the rest were rated as high risk [40, 42, 43], except for one that was rated as uncertain [38].

### Hospitalization outcome

**Fig. 3:**
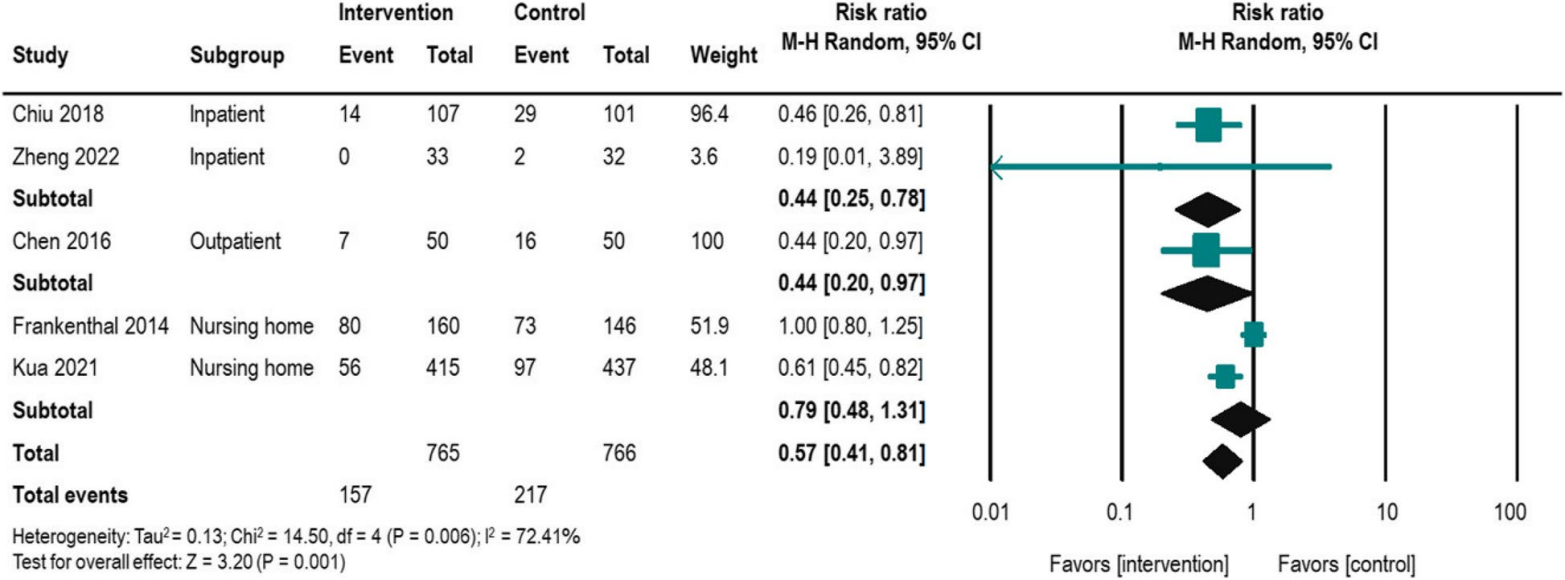
Forest plot for impact of pharmacist intervention on hospitalization

Hospitalization rates were evaluated in three RCTs and two non-RCTs. The combined sample size of the studies was 1,531 participants. A meta-analysis was performed using the outcome data (Table 2) and Fig. 3 shows a forest plot including all the studies. Although there was high heterogeneity (I^2^ = 72.41%), there was a significant improvement in hospitalization rates (*n* = 5, risk ratio = 0.57, 95% CI = 0.41–0.81). We conducted subgroup analyses to identify the settings (inpatient, outpatient, nursing home) where the pharmacist intervention yielded greater effectiveness. Although interpretation should be cautious due to small numbers in each group, we found reductions in hospitalization rates for inpatients and outpatients, and a trend toward reductions in nursing homes, but not significant. We conducted sensitivity analysis to assess the impact of study with high-risk bias study [40] on the result direction. Sensitivity analysis did not change the effectiveness of the pharmacist intervention in reducing hospitalization rates. Pharmacist intervention activity is summarized in Table 3. Three studies [40, 44, 45] utilized comprehensive medication review (CMR3), while another three [39, 44, 45] employed pharmacist intervention within a multidisciplinary team (MDT) care approach.

### Mortality outcome

**Fig. 4:**
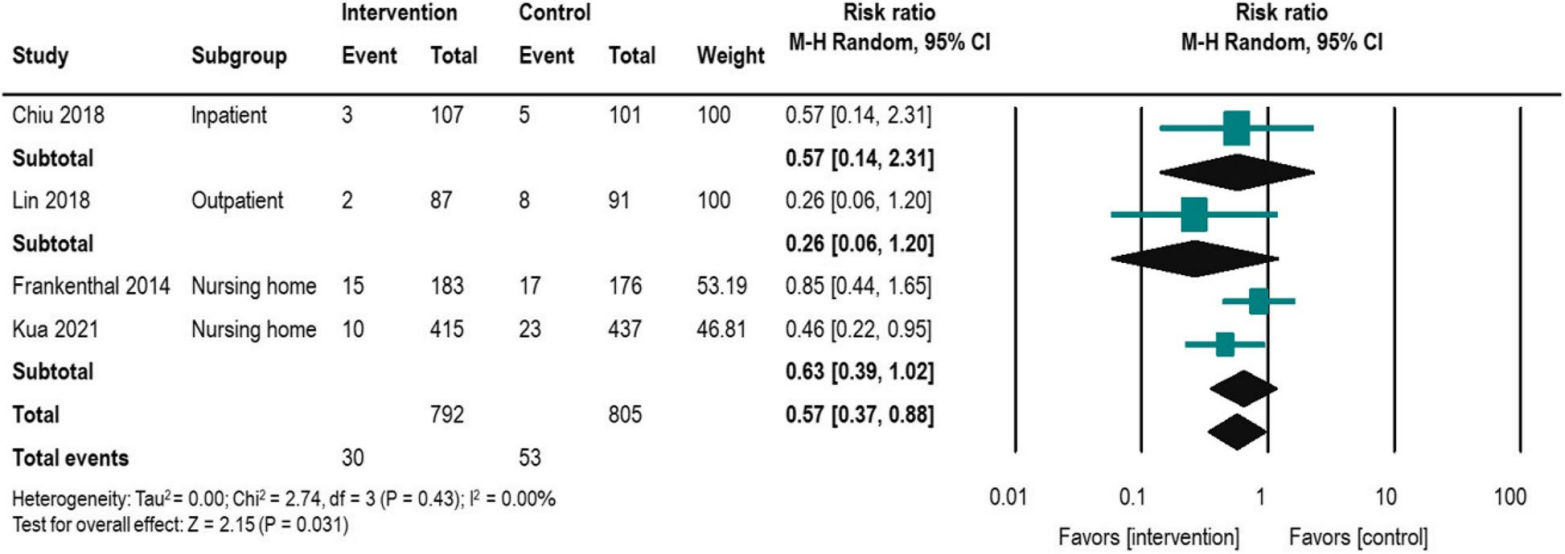
Forest plot for impact of pharmacist intervention on mortality

Mortality was evaluated in three RCTs and one non-RCT, with a total sample size of 1,597 participants, as shown in Table 2 and Fig. 4. The analysis revealed that pharmacist interventions significantly reduced the mortality rate (*n* = 4, risk ratio = 0.57, 95% CI = 0.37–0.88). There was no heterogeneity among the studies, as indicated by an I^2^ value of 0%. Subgroup analyses were conducted for mortality rates, showing a trend towards reduced mortality across all institutions (inpatient, outpatient, nursing home). However, these results lacked significance and warrant cautious interpretation due to the small sample size. Three [40, 44, 46] out of four studies conducted CMR3, while two [44, 46] involved MDT care incorporating pharmacists, and one [44] focused on deprescribing (Table 3).

### QoL outcome

**Fig. 5:**
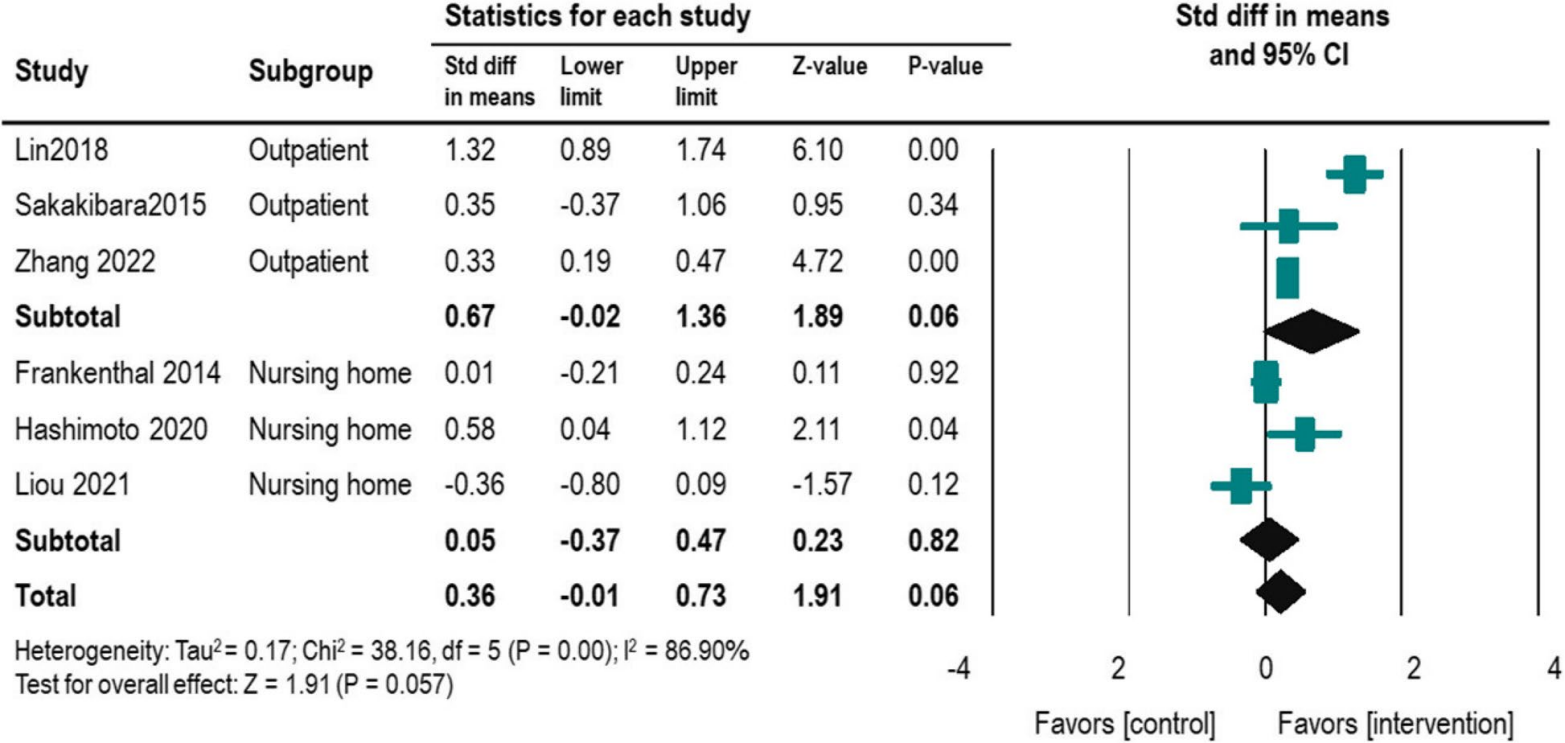
Forest plot for impact of pharmacist intervention on QoL

The impact of pharmacist interventions on QoL was assessed in three RCTs and three non- RCTs, with a total sample size of 1,083 participants. Two main types of QoL measurement tools were used: a 12-item short-form health survey (SF-12) and the European Quality of Life-5 Dimensions (EQ-5D). The EQ-5D consists of the EQ-5D descriptive system and the EQ-5D visual analogue scale (EQ-VAS). Pharmacist intervention tended to improve QoL (*n* = 6, SMD = 0.36, *P* = 0.06) (Fig. 5). The I^2^ value, which indicates heterogeneity among the included studies, was found to be very high (86.90%). When subgroup analyzed by outpatient and nursing home, pharmacist interventions showed potential for enhancing the QoL among elderly patients in outpatient settings yet yielded no significant effect in nursing homes. There were three studies [38, 43, 46] of CMR3, and two studies [43, 46] of MDT care and one [44] focused on deprescribing (Table 3).

### Certainty of evidence

GRADE assessment is shown in Table 4. The certainty of evidence was very low for QoL and low for hospitalization and mortality because of risk of bias.

**Table 4:** Certainty of evidence

**Outcome**	**Number of study**	**Risk of bias**	**Inconsistency**	**Imprecision**	**Indirectness**	**Publication bias**	**Certainty**
Hospitalization	4 RCTs, 1 non-RCT	Serious	Serious	Not serious	Not serious	Undetected	Low
Mortality	3 RCTs, 1 non-RCT	Very serious	Not serious	Not serious	Not serious	Undetected	Low
QoL	3 RCTs, 3 non-RCTs	Very serious	Very serious	Serious	Not serious	Undetected	Very low

*Abbreviations*: *RCT* randomized controlled trial, *non-RCT* non-randomized controlled trial

## Discussion

The role of pharmacists in the healthcare for older people has prompted numerous meta-analyses, yet data on patient-related outcomes remain insufficient. Ten meta-analyses on pharmacist-led interventions for older patients have been reported, with a focus on hospitalization rates [8, 21, 23–28, 48], mortality rates [21, 25, 27, 28], and QoL [28, 29]. Notably, few meta-analyses have explored the clinical outcomes of pharmacist interventions in Asian countries, which represent more than 60% of the world's population [1]. Pharmacist interventions seem effective in lowering hospitalization rates in older people, despite significant heterogeneity. Also, our study showed reduction in mortality but less distinct improvements in QoL.

A meta-analysis of five studies on hospitalization revealed a reduction in hospitalization rates (risk ratio = 0.57; 95% CI = 0.41–0.81; *P* = 0.001) among older patients residing in China [39], Hong Kong SAR [40], Israel [41], Singapore [44], and Taiwan [45]. Studies employing a comprehensive medication review (CMR3) showed notably consistent reductions in hospitalizations across different settings such as inpatients [39, 40], outpatients [45], and nursing homes [44], except for one in a nursing home with a different review type based solely on the prescription (CMR1) [41]. Our meta-analysis results support the notion that cointerventions involving CMR3 with multidisciplinary team (MDT) care or patient education may be effective at reducing hospitalizations among older patients, aligning with prior findings [8, 26]. Previous meta-analyses have shown significant reductions in the hospitalizations of older patients treated CMR3 [8] or MDT care involving pharmacists [26]. A recently published network meta-analysis study [49] demonstrated that the combination of medication review with medication reconciliation, patient education, medical staff education, and transitional care can reduce geriatric readmission rates. Several recent reports have also highlighted the efficacy of CMR3, MDT care, and medication review with various interventions as key strategies for reducing hospitalizations in older patients [8, 26, 49].

Our meta-analysis of four selected studies revealed a significant reduction in mortality among older patients residing in Asia (risk ratio = 0.57; 95% CI = 0.37–0.88; *P* = 0.031), indicating the positive impact of pharmacist interventions in this population. Comprehensive medication reviews (CMR3) [40, 44, 46] were more effective than simple prescription reviews (CMR1) [41] in reducing hospitalization rates and showed similar trends in reducing mortality. Notably, cointerventions involving CMR3 and deprescribing through MDT care in nursing homes significantly reduced mortality [44]. This finding are consistent with those of a meta-analysis by Kua et al. who demonstrated a significant reduction in mortality (odds ratio = 0.74, 95% CI = 0.65 to 0.84, *P* = 0.000) in older patients in nursing homes via medication review-directed deprescribing [11]. However, pharmacist services have been shown to be either effective or ineffective at reducing mortality [22, 27] indicating uncertainty regarding the effectiveness of medication optimization through pharmacist services, prescription reviews only (CMR1), staff education, or MDT meetings for reducing mortality among nursing home residents. To date, only four meta-analytical studies have investigated the effect of pharmacist-led interventions on mortality, and the results were not significant [21, 25, 27, 28]. Furthermore, no study has specifically examined the effects of different types of pharmacist interventions on mortality as the primary outcome. Hence, more randomized controlled trials (RCTs) with a substantial participant pool are necessary, prioritizing mortality as the primary outcome, to precisely ascertain the impact of pharmacist interventions.

Regarding QoL outcomes, the studies included in this meta-analysis employed different measurement tools. Given the limited number of selected papers, we merged the SF-12 and EQ-5D tools using SMDs, as it is not uncommon to combine different QoL assessment tools in meta-analytic studies [28, 50, 51]. The results indicated that pharmacist intervention tended to improve QoL (*n* = 6, SMD = 0.36, 95% CI = -0.01–0.73, *P* = 0.057, Fig. 3). Although the meta-analysis did not demonstrate a significant improvement, some studies reported moderate enhancements. The heterogeneity of the measurement tools and the limited number of selected papers hindered a more detailed analysis. Among the studies included in our analysis, two studies conducted in a nursing home in Taiwan [47] and Israel [41] reported no positive effect on QoL when implementing CMR1. Specifically, a study conducted in Israel showed that pharmacists' participation in a medication review based on the STOPP/START criteria had no effect on QoL (SMD = 0.01) [41], which aligns with the findings of a meta-analysis study by Taylor et al. [52]. In a study conducted in Japan, pharmacist intervention in polypharmacy deprescribing among patients with dementia resulted in a reduction in excessive sedative use and a moderate improvement in QoL, although these effects did not reach significance due to an increase in anxiety and depression [42]. Significant improvements in QoL were observed in an RCT study in Taiwan (SMD = 1.32, *P* = 0.000) [46] and two non-RCT studies in China (SMD = 0.33, *P* = 0.000) [38] and Japan (SMD = 0.58, *P* = 0.035) [43]. These studies involved CMR3 with or without MDT care as common types of pharmacist intervention, suggesting that these interventions may contribute to improving QoL and reducing hospitalization and mortality. This implication is further supported by a previous meta-analysis that indicated a positive effect of pharmacist interventions on medication adherence and QoL (*n* = 11, SMD = 0.295, *P* = 0.022), particularly when CMR3 and MDT care were implemented [28]. Meanwhile, subgroup analysis suggests that pharmacist intervention may be more effective in outpatients compared to nursing home residents on QoL. Therefore, future studies focusing on outpatients undergoing comprehensive medication reviews (CMR3) and team-based care activities (MDT care) may offer a clearer understanding of the impact of pharmacist intervention.

This study has several limitations. Studies written in the national languages of Asian countries were not included, resulting in a lack of diversity and a limited generalizability of the findings to the entire region in Asia. We tried to find journals published in Korea, but we were unable to include databases operated by Asian countries other than Korea, which is a limitation. Attempts to reduce the heterogeneity of QoL through sensitivity analysis were unsuccessful. Certainty of evidence were accessed low or very low. The low quality of evidence is attributed to studies with a high risk of bias. While many meta-analyses focus solely on RCTs, this study incorporated multiple study designs due to the limited number of eligible studies. Some meta-analyses incorporate multiple study designs in their analysis. For instance, a previous study on healthcare for older patients conducted by US pharmacists [26] and another study on the effectiveness of pharmacist services for nursing home patients employed a meta-analysis that combined various study designs [27].

The dissemination of our meta-analysis study findings holds the promise of offering new and valuable perspectives to the healthcare landscape in Asia. Furthermore, in light of the deficit in regional research, we anticipate an expansion of studies focusing on pharmacist interventions within countries sharing similar cultural contexts or even on a country-specific level. This expansion presents a unique opportunity to compare and analyze our research outcomes, fostering a deeper understanding and generating critical insights.

### Implication and practice

It comes as no surprise that pharmacists are pivotal in elderly healthcare. This study underscores the significance of pharmacist collaboration and communication with physicians, nurses, and patients for comprehensive medication reviews. Moreover, future research should delve into the efficacy of deprescribing interventions in polypharmacy. Beyond medication adherence and inappropriate medication identification, there's a pressing need to explore their impact on clinical outcomes like hospitalization, mortality, and quality of life in older patients.

## Conclusion

Our study provides evidence supporting the effectiveness of pharmacist-led interventions in reducing hospitalization and mortality rates among older patients in Asian countries. This emphasizes the need for active pharmacist participation in the healthcare system for older patients in Asia. However, our analysis did not reveal a significant improvement in QoL, indicating that further research and collaborative efforts are necessary to explore additional strategies and interventions that can enhance the QoL of older people. These findings highlight the crucial role that pharmacists can play in addressing the healthcare needs of aging populations in Asia and emphasize the importance of their active involvement in providing comprehensive care and support to older patients.

### Supplementary Information

Supplementary Material 1.

Supplementary Material 2.

Supplementary Material 3.

## Data Availability

The data that support the findings of this study are available from the corresponding author upon reasonable request.

## Abbreviations

ADE: Adverse drug event
C: Control group
CMR 1,2,3: Clinical medication review type1,2,3
CENTRAL: Cochrane central register of controlled trials
DM: Diabetes mellitus
DRP: Drug-related problem
ED: Emergency department
EQ-VAS: Europol visual analogous scale
EQ-5D: Europol scale for five domains
GRADE: Grading of Recommendations, Assessment, Development and Evaluation
HRQoL: Health-related quality of life
I: Intervention group
KMBASE: Korean medical database
MDT: Multidisciplinary team
MR: Medication reconciliation
NR: Not reported
QoL: Quality of life
RCT: Randomized controlled trial
RISS: Research information sharing service
RoB: Risk of bias criteria
RoBANS: Non-rct studies by the risk of bias assessment tool
SD: Standard deviation
SF-12: 12-Item short-form health survey
SMD: Standardized mean difference
START: Screening tool to alert doctors to right treatment
STOPP: Screening tool of older person’s prescriptions
UK: United Kingdom
US: United states

## References

[1] World population prospects 2022 : Summary of Results New York: Department of Economic and Social Affairs Population Division 2022 [Available from: https://reliefweb.int/report/world/world-population-prospects-2022-summary-results .

[2] Bhagavathula AS, Gebreyohannes EA, Fialova D (2022). Prevalence of polypharmacy and risks of potentially inappropriate medication use in the older population in a developing country: a systematic review and meta-analysis. Gerontology.

[3] Chang TI, Park H, Kim DW, Jeon EK, Rhee CM, Kalantar-Zadeh K (2020). Polypharmacy, hospitalization, and mortality risk: a nationwide cohort study. Sci Rep.

[4] Tian F, Chen Z, Wu J (2022). Prevalence of polypharmacy and potentially inappropriate medications use in elderly chinese patients: a systematic review and meta-analysis. Front Pharmacol.

[5] Cossette B, Ethier JF, Joly-Mischlich T, Bergeron J, Ricard G, Brazeau S (2017). Reduction in targeted potentially inappropriate medication use in elderly inpatients: a pragmatic randomized controlled trial. Eur J Clin Pharmacol.

[6] Kurczewska-Michalak M, Lewek P, Jankowska-Polanska B, Giardini A, Granata N, Maffoni M (2021). Polypharmacy management in the older adults: a scoping review of available interventions. Front Pharmacol.

[7] Clyne W, Blenkinsopp A, Seal R. A guide to medication review. Liverpool: The National prescribing cnetre (NPC); 2008. Available from: http://hdl.handle.net/10026.1/16326 .

[8] Mizokami F, Mizuno T, Kanamori K, Oyama S, Nagamatsu T, Lee JK (2019). Clinical medication review type III of polypharmacy reduced unplanned hospitalizations in older adults: A meta-analysis of randomized clinical trials. Geriatr Gerontol Int.

[9] Scott IA, Hilmer SN, Reeve E, Potter K, Le Couteur D, Rigby D (2015). Reducing inappropriate polypharmacy: the process of deprescribing. JAMA Intern Med.

[10] Bloomfield HE, Greer N, Linsky AM, Bolduc J, Naidl T, Vardeny O (2020). Deprescribing for community-dwelling older adults: a systematic review and meta-analysis. J Gen Intern Med.

[11] Kua CH, Mak VSL, Huey Lee SW. Health Outcomes of Deprescribing Interventions Among Older Residents in Nursing Homes: A Systematic Review and Meta-analysis. J Am Med Dir Assoc. 2019;20(3):362–72e11. 10.1016/j.jamda.2018.10.026 30581126

[12] Thillainadesan J, Gnjidic, D., Green, S., & Hilmer, S. N. . Impact of Deprescribing Interventions in Older Hospitalised Patients on Prescribing and Clinical Outcomes: A Systematic Review of Randomised Trials. Drugs Aging. 2018;35(4):303–19. 10.1007/s40266-018-0536-4 29541966

[13] Geurts MM, Talsma J, Brouwers JR, de Gier JJ (2012). Medication review and reconciliation with cooperation between pharmacist and general practitioner and the benefit for the patient: a systematic review. Br J Clin Pharmacol.

[14] Cho HJ, Chae J, Yoon SH, Kim DS (2022). Aging and the Prevalence of polypharmacy and hyper-polypharmacy among older adults in south korea: a national retrospective study during 2010–2019. Front Pharmacol.

[15] Lee SWH, Bell JS, van Mil JWF, Alvarez-Risco A (2019). Pharmaceutical Care in Asia. Alves da Costa F.

[16] Maria JL, Anand TN, Dona B, Prinu J, Prabhakaran D, Jeemon P (2021). Task-sharing interventions for improving control of diabetes in low-income and middle-income countries: a systematic review and meta-analysis. Lancet Glob Health.

[17] Flood D, Hane J, Dunn M, Brown SJ, Wagenaar BH, Rogers EA (2020). Health system interventions for adults with type 2 diabetes in low- and middle-income countries: A systematic review and meta-analysis. PLoS Med.

[18] Ogedegbe G, Gyamfi J, Plange-Rhule J, Surkis A, Rosenthal DM, Airhihenbuwa C (2014). Task shifting interventions for cardiovascular risk reduction in low-income and middle-income countries: a systematic review of randomised controlled trials. BMJ Open.

[19] Rawal KB, Mateti UV, Shetty V, Shastry CS, Unnikrishnan MK, Shetty S (2023). Development of evidence-based indicators for the detection of drug-related problems among ovarian cancer patients. Front Pharmacol.

[20] Verma A, Saha S, Jarl J, Conlon E, cGuinness B, Trépel D. An Overview of Systematic Reviews and Meta-Analyses on the Effect of Medication Interventions Targeting Polypharmacy for Frail Older Adults. J Clin Med. 2023;12(4):1379. 10.3390/jcm12041379 PMC9960328 36835915

[21] Abbott RA, Moore DA, Rogers M, Bethel A, Stein K, Coon JT (2020). Effectiveness of pharmacist home visits for individuals at risk of medication-related problems: a systematic review and meta-analysis of randomised controlled trials. BMC Health Serv Res.

[22] Almutairi H, Stafford A, Etherton-Beer C, Flicker L (2020). Optimisation of medications used in residential aged care facilities: a systematic review and meta-analysis of randomised controlled trials. BMC Geriatr.

[23] Tasai S, Kumpat N, Dilokthornsakul P, Chaiyakunapruk N, Saini B, Dhippayom T (2019). Impact of medication reviews delivered by community pharmacist to elderly patients on polypharmacy: a meta-analysis of randomized controlled trials. J Patient Saf.

[24] Thomas R, Huntley AL, Mann M, Huws D, Elwyn G, Paranjothy S (2014). Pharmacist-led interventions to reduce unplanned admissions for older people: a systematic review and meta-analysis of randomised controlled trials. Age Ageing.

[25] Holland R, Desborough J, Goodyer L, Hall S, Wright D, Loke YK (2008). Does pharmacist-led medication review help to reduce hospital admissions and deaths in older people? A systematic review and meta-analysis. Br J Clin Pharmacol.

[26] Lee JK, Slack MK, Martin J, Ehrman C, Chisholm-Burns M. Geriatric patient care by U.S. pharmacists in healthcare teams: systematic review and meta-analyses. J Am Geriatr Soc. 2013;61(7):1119–27. 10.1111/jgs.12323 23796001

[27] Lee SWH, Mak VSL, Tang YW (2019). Pharmacist services in nursing homes: A systematic review and meta-analysis. Br J Clin Pharmacol.

[28] Marcum ZA, Jiang S, Bacci JL, Ruppar TM (2021). Pharmacist-led interventions to improve medication adherence in older adults: A meta-analysis. J Am Geriatr Soc.

[29] Loh ZW, Cheen MH, Wee HL (2016). Humanistic and economic outcomes of pharmacist-provided medication review in the community-dwelling elderly: A systematic review and meta-analysis. J Clin Pharm Ther.

[30] Page MJ, McKenzie JE, Bossuyt PM, Boutron I, Hoffmann TC, Mulrow CD (2021). The PRISMA 2020 statement: an updated guideline for reporting systematic reviews. BMJ.

[31] Grimes DA, Schulz KF (2002). An overview of clinical research: the lay of the land. Lancet.

[32] SDG indicators. Regional groupings used in Report and Statistical Annex. Sustainable Development Goals of the United Nations (online). 2022 [Available from: https://unstats.un.org/sdgs/indicators/regional-groups/ .

[33] Sterne JA, Hernan MA, Reeves BC, Savovic J, Berkman ND, Viswanathan M (2016). ROBINS-I: a tool for assessing risk of bias in non-randomised studies of interventions. BMJ.

[34] Sterne JAC, Savovic J, Page MJ, Elbers RG, Blencowe NS, Boutron I (2019). RoB 2: a revised tool for assessing risk of bias in randomised trials. BMJ.

[35] J. C. Statistical Power Analysis for the Behavioral Sciences. 2nd Ed. ed: Hillsdale, NJ: Lawrence Erlbaum Associates; 1988.

[36] Cochrane Handbook for Systematic Reviews of Interventions version 6.4 (updated August 2023). Cochrane, 2023. 2022. Available from: www.training.cochrane.org/handbook .

[37] Guyatt GH, Oxman AD, Vist GE, Kunz R, Falck-Ytter Y, Alonso-Coello P (2008). GRADE: an emerging consensus on rating quality of evidence and strength of recommendations. BMJ.

[38] Zhang S, Zhu D, Qi Z, Tian L, Qian S, Song D, et al. Effects of home medication review on drug-related problems and health-related quality of life among community-dwelling older adults in China. J Am Pharm Assoc (2003). 2022;62(2):481–6. 10.1016/j.japh.2021.10.023 34776338

[39] Zheng X, Xiao L, Li Y, Qiu F, Huang W, Li X (2022). Improving safety and efficacy with pharmacist medication reconciliation in orthopedic joint surgery within an enhanced recovery after surgery program. BMC Health Serv Res.

[40] Chiu KC, Lee WK, See YW, Chan HW (2018). Outcomes of a pharmacist-led medication review programme for hospitalised elderly patients. Hong Kong Med J.

[41] Frankenthal D, Lerman Y, Kalendaryev E, Lerman Y (2014). Intervention with the screening tool of older persons potentially inappropriate prescriptions/screening tool to alert doctors to right treatment criteria in elderly residents of a chronic geriatric facility: a randomized clinical trial. J Am Geriatr Soc.

[42] Sakakibara M, Igarashi A, Takase Y, Kamei H, Nabeshima T (2015). Effects of prescription drug reduction on quality of life in community-dwelling patients with dementia. J Pharm Pharm Sci.

[43] Hashimoto R, Fujii K, Shimoji S, Utsumi A, Hosokawa K, Tochino H (2020). Study of pharmacist intervention in polypharmacy among older patients: Non-randomized, controlled trial. Geriatr Gerontol Int.

[44] Kua CH, Yeo CYY, Tan PC, Char CWT, Tan CWY, Mak V, et al. Association of Deprescribing With Reduction in Mortality and Hospitalization: A Pragmatic Stepped-Wedge Cluster-Randomized Controlled Trial. J Am Med Dir Assoc. 2021;22(1):82–9e3. 10.1016/j.jamda.2020.03.012 32423694

[45] Chen JH, Ou HT, Lin TC, Lai EC, Kao YH (2016). Pharmaceutical care of elderly patients with poorly controlled type 2 diabetes mellitus: a randomized controlled trial. Int J Clin Pharm.

[46] Lin HW, Lin CH, Chang CK, Chou CY, Yu IW, Lin CC (2018). Economic outcomes of pharmacist-physician medication therapy management for polypharmacy elderly: A prospective, randomized, controlled trial. J Formos Med Assoc.

[47] Liou W-S, Huang S-M, Lee W-H, Chang Y-L, Wu M-F. The effects of a pharmacist-led medication review in a nursing home. Medicine. 2021;100(48):e28023. 10.1097/MD.0000000000028023 PMC9191564 35049214

[48] Christopher CM, Kc B, Blebil A, Alex D, Ibrahim MIM, Ismail N, et al. Clinical and Humanistic Outcomes of Community Pharmacy-Based Healthcare Interventions Regarding Medication Use in Older Adults: A Systematic Review and Meta-Analysis. Healthcare (Basel). 2021;9(11):1577. 10.3390/healthcare9111577 PMC8625440 34828622

[49] Dautzenberg L, Bretagne L, Koek HL, Tsokani S, Zevgiti S, Rodondi N (2021). Medication review interventions to reduce hospital readmissions in older people. J Am Geriatr Soc.

[50] Lu S, Zhang AY, Liu T, Choy JCP, Ma MSL, Wong G (2021). Degree of personalisation in tailored activities and its effect on behavioural and psychological symptoms and quality of life among people with dementia: a systematic review and meta-analysis. BMJ Open.

[51] Rahja M, Laver K, Whitehead C, Pietsch A, Oliver E, Crotty M. A systematic review and meta-analysis of reablement interventions for people in permanent residential aged care homes. Age Ageing. 2022;51(10):afac208. 10.1093/ageing/afac208 36215172

[52] Hill-Taylor B, Walsh KA, Stewart S, Hayden J, Byrne S, Sketris IS (2016). Effectiveness of the STOPP/START (Screening Tool of Older Persons' potentially inappropriate Prescriptions/Screening Tool to Alert doctors to the Right Treatment) criteria: systematic review and meta-analysis of randomized controlled studies. J Clin Pharm Ther.

