# Supplementary material for: Pharmacist interventions in Asian healthcare environments for older people: a systematic review and meta-analysis on hospitalization, mortality, and quality of life
Source: BMC Geriatr. 2024 Jun 12;24:513. doi: 10.1186/s12877-024-05089-9 (PMC11170896; doi:10.1186/s12877-024-05089-9)
Supplement: Supplementary file 3 — Supplementary Material 3. [file 12877_2024_5089_MOESM3_ESM.docx]

**Search Strategy**

1. **PubMed** (June 30, 2023): (("older adults"[Title/Abstract] OR "old peo-ple"[Title/Abstract] OR old*[Title/Abstract] OR "older people"[Title/Abstract] OR "older persons"[Title/Abstract] OR elder*[Title/Abstract] OR senior[Title/Abstract] OR frail[Title/Abstract] OR "aged elderly"[Title/Abstract] OR aged[Title/Abstract] OR ger-iatric[Title/Abstract] OR "geriatric patient"[Title/Abstract] OR "elderly pa-tient"[Title/Abstract]) AND pharmacist* [Title/Abstract])
2. **Cochrane** (June 30, 2023): (("older adults" OR "old people" OR old* OR "old-er people" OR "older persons" OR elder* OR senior OR frail OR "aged elderly" OR aged OR geriatric OR "geriatric patient" OR "elderly patient") and pharmacist)
3. **EMBASE** (June 30, 2023): 'aged'/exp AND 'pharmacist':ti
4. **KMBASE** (June 30, 2023): ((([ALL=elderly] OR [ALL=geriatric]) OR [ALL=aged]) AND [ALL=pharmacist])
5. RISS (June 30, 2023): elderly and pharmacist
